# Transhepatic ultrasound guided embolization as a successful novel technique in treatment of pediatric complex intrahepatic arterioportal fistula: a case report and review of the literature

**DOI:** 10.1186/s13256-023-04047-0

**Published:** 2023-09-15

**Authors:** Heba Taher, ElSayed Kidr, Ahmed Kamal, Mohamed ElGobashy, Shady Mashhour, Amr Nassef, Sherifa Tawfik, Gamal El Tagy, Muayad Shaban, Haytham Eltantawi, Khaled S. Abdullateef

**Affiliations:** 1 Pediatric Surgery Department, Specialized Pediatric Hospital, Cairo University Kasr Al Einy, Faculty of Medicine, 1 Abou El Rish Sq., El Sayeda Zeinab, Cairo, Egypt; 2 Department of Radiology, Cairo University Kasr Al Einy, Faculty of Medicine, Cairo, Egypt; 3 Pathology Department, Ministry of Health, Cairo, Egypt

**Keywords:** Intrahepatic vascular shunts, Portal hypertension, Congenital arterioportal fistula, Trans hepatic embolization, Transarterial embolization

## Abstract

**Introduction:**

Intrahepatic vascular shunts “IHVS” are abnormal communications between intra-hepatic vasculature involving the arterial, portal, or hepatic venous system. Arterio-portal fistula “APF” is an intrahepatic communication between the hepatic arterial system and the portal venous system without any communication with the systemic venous circulation. APF is considered a rare cause of portal hypertension and gastrointestinal bleeding in infancy.

**Case presentation:**

A 3-month-old Mediterranean female with known cardiac congenital anomalies presented to us with abdominal distension and diarrhea. Ultrasonography revealed massive ascites and computerized tomography (CT) abdomen with intravenous (IV) contrast revealed a left hepatic lesion. On further evaluation, an intrahepatic arterio-portal vascular malformation was detected. Attempted trans arterial embolization failed and radiology team successfully carried out direct trans hepatic ultrasound guided coiling of the aneurysmal venous sac followed by successful resection of segment 4 of the liver with the vascular malformation avoiding life threatening intra operative bleeding.

**Conclusion:**

Any child with recurrent gastrointestinal bleeding, failure to thrive, vomiting, diarrhea, steatorrhea, splenomegaly, or ascites should be investigated for intrahepatic arterio-portal fistula “IAPF”. Our novel technique of direct trans hepatic ultrasound guided coiling is an alternative method if trans arterial embolization “TAE” failed.

## Introduction

Arterio-portal fistula “APF” is an intrahepatic communication between the hepatic arterial system and the portal venous system without any communication with the systemic venous circulation. The etiology of APF may be congenital “Primary” or acquired “Secondary”. The acquired form is most often due to trauma [1], surgical procedures [2–4] or rupture hepatic artery aneurism [5], transhepatic intervention [6] or biopsy [7, 8]. On the other hand, congenital arterioportal fistulae are rare, with only a small number of case reports describing this entity in children—44 cases—we report here the 45th case of a congenital APF which is a complex type of IHAPF and treated with a Novel technique due to failure of intrahepatic arterial embolization. Surgical treatment was not considered the first option because of the aberrant vasculature and the major concern about uncontrolled intraoperative bleeding.

This case is unique because it highlights the importance of preoperative radiological interventions preoperative to reduce risks of fatal intraoperative bleeding, even in failed attempts of radiological interventions there are novel techniques to achieve ideal preoperative control.

## Case presentation

A 3-month-old Mediterranean female, full term after cesarean section “CS”, Apgar score of 8 which was normal and normal birth weight. with cardiac anomalies in the form of atrial septal defect “ASD”—3 × 3.5 ml—and patent foramen ovale “PDA” -closed spontaneously-, presented with failure to thrive, abdominal distention and diarrhea. Physical examination showed hepatomegaly and superficial dilated veins which are suggestive of portal hypertension. Ultrasound revealed massive ascites.

On at admission the child weight was 2.5 kg and length 49 cm both were on the low centile for age.

There was negative family history of medical diseases and negative consanguinity hemoglobin was 8.6 g/dl, albumin was 2.8 g/dl, and prealbumin 13.6 mg/dl. Laboratory values were white blood cell count 10.6 × 10^3^/mm^3^, platelet count 500 × 10^3^/mm^3^, normal prothrombin and partial thromboplastin times, serum sodium 120 mmol/l, potassium 6.2 mmol/l, chloride 80 mmol/l, CO_2_ 22 mmol/l, urea 26 mg/dl, creatinine 0.5 mg/dl, aspartate aminotransferase 105 U/l, alanine aminotransferase 65 U/l, alkaline phosphatase 248 U/l, γ-glutamyl transferase 167 U/l, total bilirubin 0.8 mg/dl.

**Fig. 1:**
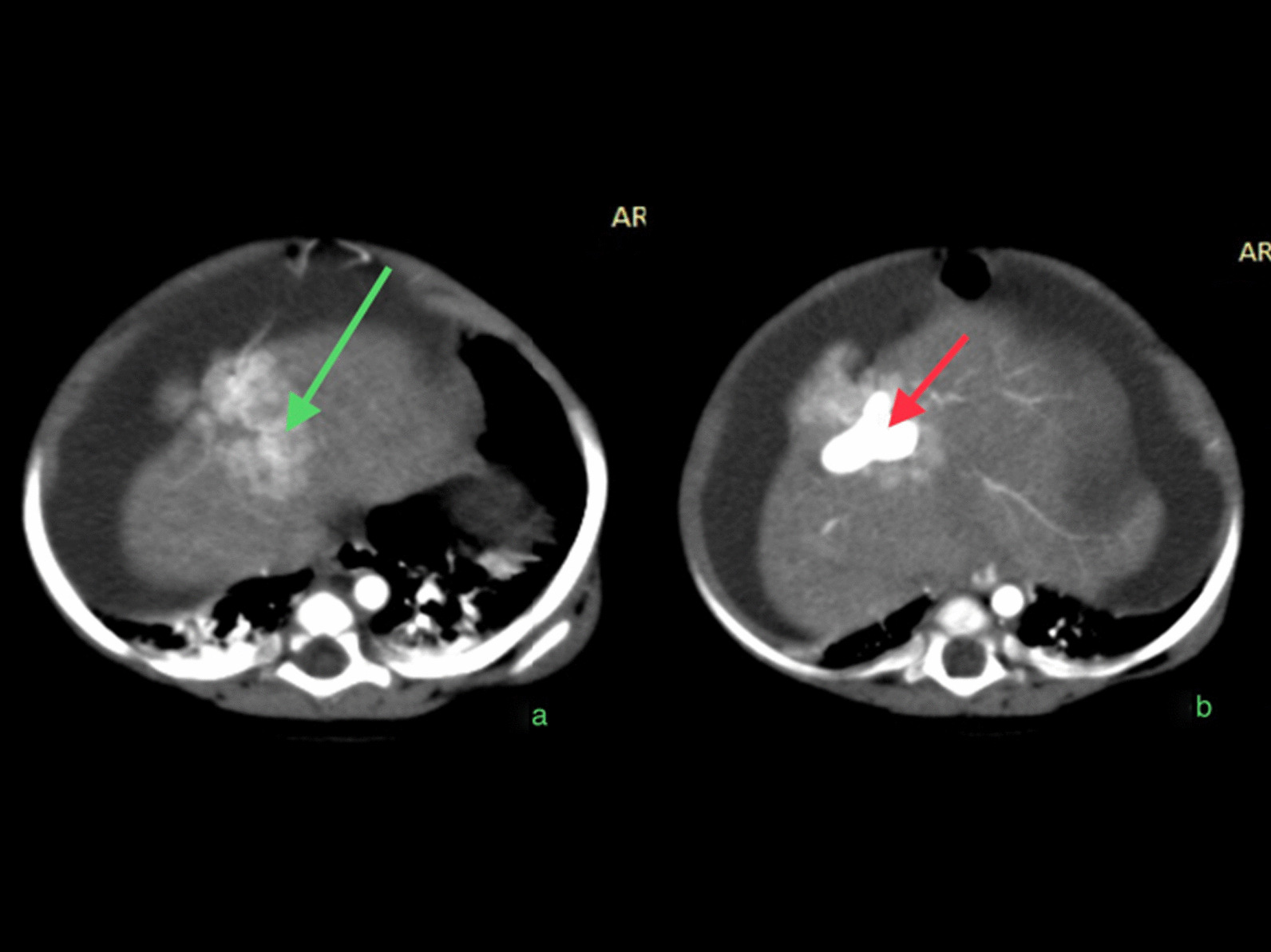
Multislice CT hepatic angiography and portography; **a** soft tissue enhancing mass (Green arrow), **b** intensely enhancing cystic lesion (vascular space) enhancing during the arterial phase vascular arterial lesion (red arrow)

**Fig. 2:**
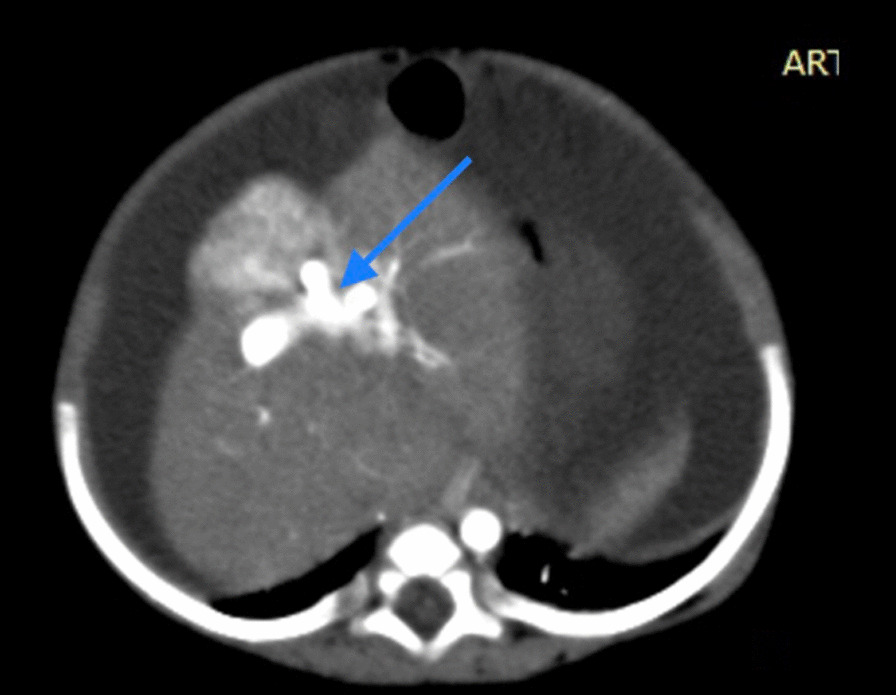
Portal phase showing early opacification of portal vein. The arrow is pointing at the portal vein on (Computerised tomography scan with intravenous contrast)

**Fig. 3:**
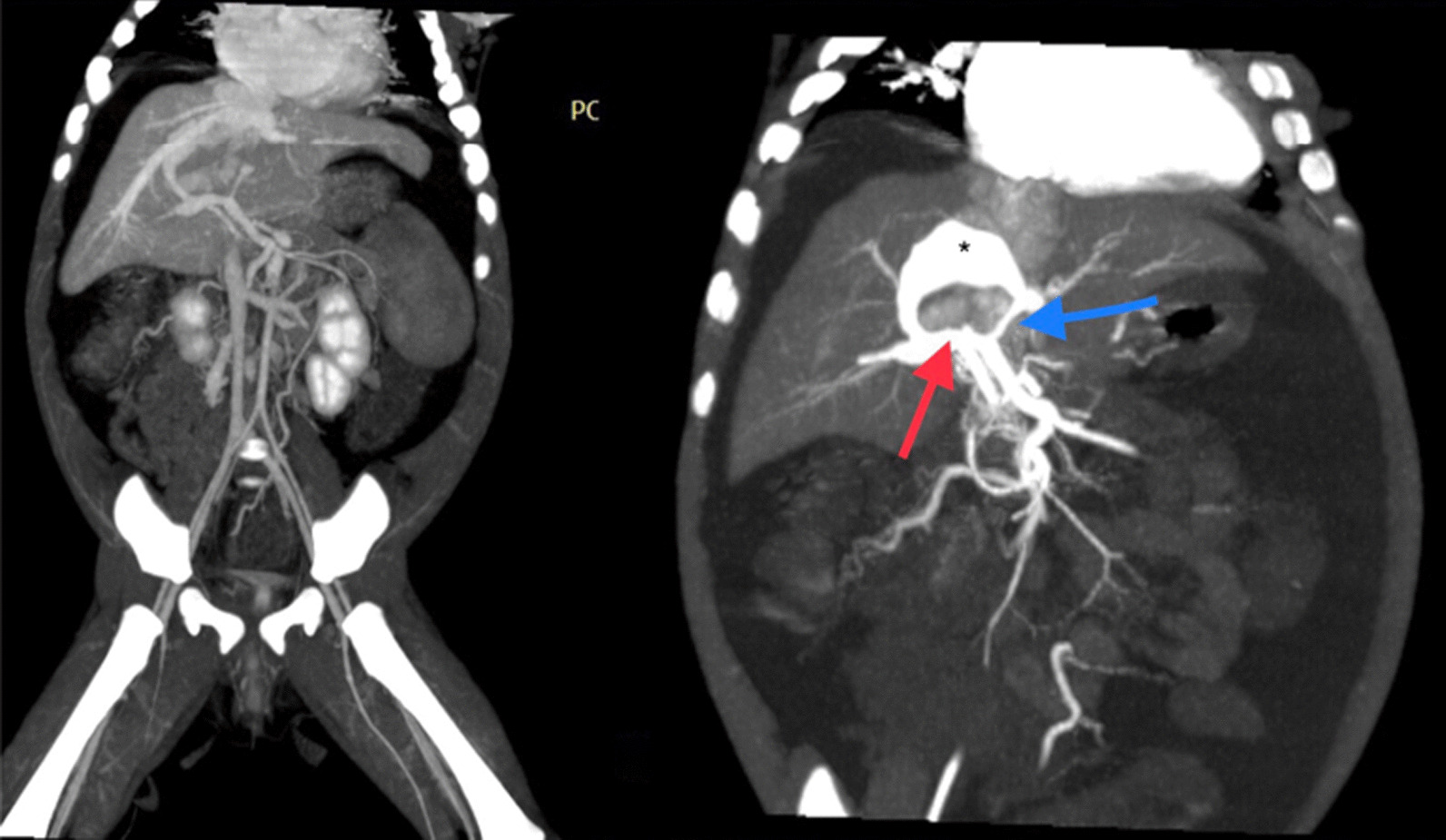
MSCT showing right aberrant hepatic artery (red arrow) communicating with vascular sac (Astrix) with communication to left portal vein (blue arrow) representing a complex intrahepatic arterioportal malformation that is no direct communication

**Fig. 4:**
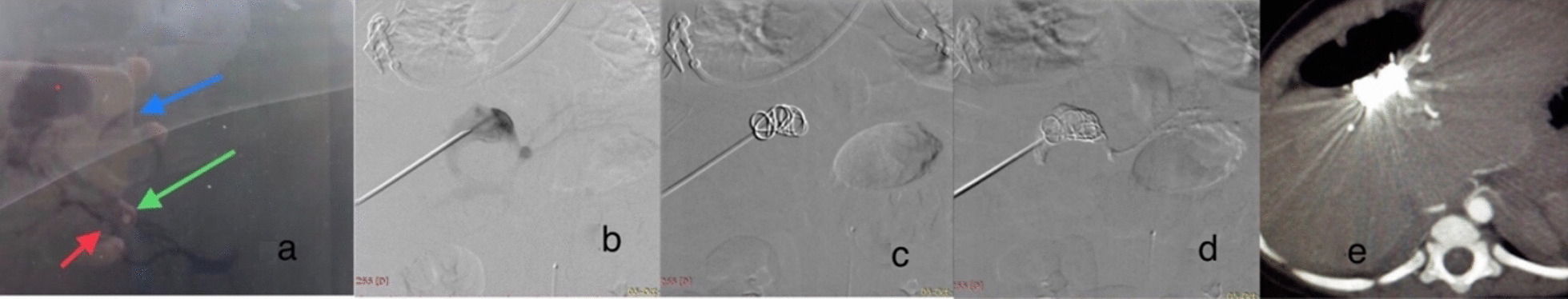
**a–d** Angiography; **a** right hepatic artery (red arrow), aberrant hepatic artery (green arrow), filling of portal vein (blue arrow); **b** direct ultrasound guided injection of glue (histoacryle) into the cystic malformation using 20 G needle and coiling (**c**). **d** controlled angiography revealed successful embolization of the vascular component of the malformation. **e** Post-operative CT appearance after resection of segment 4

CT abdomen with IV contrast was done and revealed a well-defined lesion at the left lobe of the liver, which was suspected to be hemangioendothelioma, all liver and kidney functions were normal. MSCT angiography and portography “Multi-slice computed tomography” was done and revealed complex intrahepatic arterio-portal vascular malformation (Figs. 1, 2, 3) in segment 4 between an apparent atypical branch of the hepatic artery proper and the left portal vein, the apparent branch was communicating with a dilated venous cystic structure which was communicating with left main portal vein there was an associated solid component showing up as a parenchymal stain. The patient was referred to the interventional radiology department for embolization (Fig. 4a–d) using an endovascular micro-catheter through the celiac trunk and pancreaticoduodenal arcade, but both failed which is thought to be due to hyper-dynamic circulation related thrombosis of the proper hepatic artery which was replaced by multiple collaterals.

The usual trans arterial route embolization through this complex vascularity was impossible therefore using a direct ultrasound guided using an 18G spinal needle trans hepatic obliteration of the cystic (the aneurysmal venous sac) component by histo-acryl which is a glue ratio 1:3 (histoacryl: lipiodol) and coiling as well a single coil 15 mm × 30 cm detachable coil while the second component the intraparenchymal multiple channels between the artery and vein at segment 4 was obliterated by gel foam. Follow-up controlled angiography showed that the vascular communication (cystic component) was closed. The remaining solid intraparenchymal component was surgically removed two days later by left medial segmentectomy -segment 4- since not all the channels were obliterated by gel foam (Fig. 4e).

The patient was discharged to the ICU postoperatively, then the patient was transferred to the department and then discharged with an uneventful post-operative period. After 4 months of follow-up, the portal hypertension symptoms -ascites completely resolved, but there were very small residuals for follow-up every 6 months and the patient is completely asymptomatic. Weight on follow up at 2 years of age is 16 kg which is 75th centile for age.

## Discussion

Preoperative radiologic interventions prior to liver resections with vascular malformations is necessarily to reduce risk of fatal intraoperative bleeding, but when vasculature is so aberrant and embolisation fails, novel approaches still need to be sought to reduce intraoperative risk of bleeding, reports on successful trans hepatic ultrasound guided embolisation are very few, in this case report we report a successful case which was managed preoperatively with this novel approach.

Congenital intrahepatic arterio-portal fistula “IAPF” was first reported approximately 50 years ago by Gryboski *et al.* [9] but its etiology remains unclear. Now it is defined as an intrahepatic communication between the hepatic artery and the portal venous system. IAPF is an uncommon cause of presinusoidal portal hypertension (PH) and is believed to be the result of increased blood flow in the portal system. Congenital IAPF is a rare entity and is always diffuse or multiple [10], whereas a solitary fistula is always acquired. IAPF identified in adulthood is difficult to diagnose as congenital [10]. Based on a study that was made by Vauthey *et al.* only 10% of IAPF is congenital [11]. Until now, only 45 cases -including our case- of congenital IAPF have been reported in the literature, mostly occurring in infants.

Norton-Jacobson classified IAPF (Table 1) based primarily on the observations that therapeutic occlusion is normally directed at the arterial side and that outcome after various treatments is heavily influenced by the arterial vascular anatomy [12] so they classified IAPF according to the afferent vessels supplying them into unilateral, bilateral or complex. A unilateral IAPF (type 1) is supplied by only one of the right, left, or main hepatic arteries. Bilateral lesions (type 2) include supply from both of the parent hepatic arteries or their branches. Complex lesions (type 3), as in our case, consist typically of a plexiform vascular nidus with multiple feeding arteries, including supply from arteries other than the hepatic arteries.

**Table 1:** Norton-Jacobson classification of IAPF [12]

Afferent supply	Description of afferent vessel(s)	Initial treatment
Unilateral	RHA or LHA or main HA only	TAE
Bilateral	Both RHA and LHA* or equivalent supply from main HA^†^	TAE Ligation if TAE fails
Complex	RHA and/or LHA and no hepatic arterial supply^‡^	Ligation/surgery^§^

^*^Both parent hepatic arteries and/or branches from both parent arteries

^†^Equivalent supply from the main hepatic artery may take the place of one of the parent arteries

^‡^Examples of other arteries include celiac, superior mesenteric, gastric, gastroduodenal, inferior phrenic, pancreaticoduodenal, jejunal, cystic, adrenal and splenic arteries

^§^The current case was the first case of congenital IAPF treated exclusively by TAE; thus, data are insufficient to recommend TAE as initial therapy at this time

*IAPF* intrahepatic arterioportal fistula, *RHA* right hepatic artery, *LHA* left hepatic artery, *HA* hepatic artery, *TAE* transarterial embolization(s)

Most symptoms and signs of IAPF (Table 2) are caused by the development of portal hypertension [13–16]. The commonest symptom and signs are upper GI bleeding (63% of cases), And Splenomegaly (54% of cases) In 20% (*n* = 9) of affected patients, they have trisomy 21, however, a relationship between both of them is not defined yet. The only other association as in our case was congenital heart disease (*n* = 3, 6.7%) as atrial, ventricular septal defects, and patent ductus arteriosus.

**Table 2:** Summarises the demographic data and symptoms and signs of IAPF based on reports published in literature about this condition

Clinical review (*n* = 45) [9, 11–13, 15–18, 21–23, 26–48]	Percentage (number of cases)
Gender	
Male	60% (27)
Female	40% (18)
Type	
Unilateral	44.5% (20)
Bilateral	20% (9)
Complex	29% (13)
Unreported	6.5% (3)
Mean age	
Overall	28 months (1 week–16 years)
Unilateral	46 months (3 weeks–16 years)
Bilateral	14 months (1 week–6 years)
Complex	12.5 months (2 months–6 years)
Symptoms	
Upper GI bleeding	63% (28)
Failure to thrive	53.5% (24)
Diarrhea or steatorrhea	42.2% (19)
Vomiting	18% (8)
Signs	
Splenomegaly	54% (24)
Ascites or edema	51% (23)
Abdominal distension	36% (16)
Hepatomegaly	33.5% (15)
Right upper quadrant bruit	36% (16)
Laboratory	
Anemia	73.5% (33)

Complications of IAPF include hemorrhagic shock from variceal bleeding [21], high output congestive heart failure “CHF” in very young infants [9, 11, 16, 22] because of left to right shunt through a patent ductus venosus [23], as flow is otherwise restricted by hepatic sinusoids interposed between the fistula and the right heart [1, 10, 24]. CHF occurs more commonly in hepatic arteriovenous malformation “AVM”, a communication between a systemic artery and a hepatic vein, as well as hepatic hemangiomata and hemangioendotheliomas [25], which are abnormal multiple connections between the hepatic artery and hepatic vein. Congenital IAPF differs from these lesions because it does not communicate directly with systemic venous circulation. Thrombocytopenia [21, 26], coagulopathy [21, 26], bacterial peritonitis [21] can be observed in some cases. High flow-related Thrombosis of the arterial part of the IAPF can also be a complication that may cause the failure of catheterization as observed in our case.

Laboratory investigations (Table 2) mostly show anemia in the affected children (73.5% of cases), hypoalbuminemia (16%) and occult blood in the stools (14%).

Hyperbilirubinemia has also been noted [26]. Ultrasonography is considered the first line of investigation to be done. Hepatic angiography can be done later to confirm the diagnosis and identify the vascular malformation. CT “computerized tomography” can be a useful diagnostic modality with the recent advances in radiomics [52–56] which is using algorithms and images to enhance diagnostic accuracy and differentiate from various congenital anomalies which might lead to GIT bleeding and other associated vascular malformations.

The different presentations of IAPF as illustrated before are due to the different sizes and locations of the shunt [12]. The more blood shunted, the more severe the symptoms and signs of IAPF [11]. Mesenteric vascular congestion is the reason behind gastrointestinal hemorrhage, chronic malabsorption, diarrhea, and steatorrhea [9, 12, 23, 28, 33]. Furthermore protein-losing enteropathy, steatorrhea, and fat malabsorption may occur secondary to small bowel ischemia and can lead to malnutrition and failure to thrive of the affected individuals [34, 49]. Another mechanism for fat malabsorption is hypo-pancreatism and lymphatic vessel leakage secondary to abnormal portal circulation [33, 34, 57]. With antegrade flow in the portal vein being decreased, there is an associated increase in hepatic arterial flow. It has been suggested that this results in a “steal” phenomenon with a decrease in blood flow in the aortic branches distal to the celiac trunk that led to worsening of small bowel edema and hemorrhage [16, 32, 33]. Intestinal biopsy of affected children may be normal [28] or may show some vascular dilatation, intestinal edema, and fibrosis of the lamina propria [23].

In the literature, treatment modalities of IAPF include radiological embolization, surgery (for example arterial ligation or portocaval shunt), liver resection (for example lobectomy), or liver transplantation. Combined approaches can be done as embolization and later ligation or resection.

Table 3 summarizes the intervention type and outcome of the 45 patients of IAPF in literature (Table 3), overall, 37 patients were treated primarily by embolization with a success rate of almost 65% (*n* = 24), while only 13 needed surgical intervention whether arterial ligation, portocaval shunt, liver resection or liver transplantation. Details of surgical intervention are summarized in Table 3.

**Table 3:** Summarises different management approaches of IAPF of reported in literature

Management	Overall cases, *n* = 45 (%)	Unilateral, *n* = 20 (%)	Bilateral, *n* = 9 (%)	Complex, *n* = 14 (%)	Unreported, *n* = 2 (%)
Radiological Embolization	37 (82.2%)	13 (65%)	9 (100%)	14 (100%)	1 (50%)
Success overall	24 (64.9%)	12 (92.5%)	5 (55.6%)	5 (35.7%)	1 (100%)
Need surgery	13 (35.1%)	1 (7.5%)	4 (44.4%)	9 (64.3%)	0
Surgery	7 (15.6%)	1 (5%)	2 (22.2%)	4 (28.6%)	–
Arterial ligation	6 (13.3%)	1 (5%)	2 (22.2%)	3 (21.4%)	–
Success	6 (85.7%)	0	2 (100%)	4 (100%)	–
Liver resection	6 (13.3%)	1 (5%)	2 (22.2%)	3 (21.4%)	–
Success	6 (100%)	1 (100%)	2 (100%)	3 (100%)	–
Liver transplantation	1 (2.2%)	–	–	2 (14.3%)	–
Success	1 (100%)	–	–	1 (50%), 1 (awaiting)	–
Nil	1 (2.2%)	–	–	–	1 (50%)

In unilateral IAPF: Only one case needed liver resection after the failure of arterial ligation [47]. Given this percentage, transarterial embolization “TAE” is very likely to be effective for unilateral lesions if only one feeding artery is present.

In bilateral IAPF: all t patients were initially managed by embolization with a success rate of 55.6% only (*n* = 5). Two patients were successfully managed by arterial ligation and another two went through liver resection to relieve the symptoms. Some authors [11, 50, 51] have proposed that bilateral lesions should be treated by ligation of feeding arteries, with embolization affording only short-term palliation. However, repeated endovascular interventions are often necessary to be curative, particularly if multiple bilateral feeding arteries are present or if collaterals subsequently develop [15, 18, 43].^.^

On reviewing the 14 reported cases (including our patient) of complex IAPF, only 5 cases were successfully managed by embolization (35.7%). Three patients underwent arterial ligation and another one had portportocavalnt with a success rate of 100% (*n* = 4). Another three cases were managed successfully by liver resection and the last two cases with liver transplantation.

In our patient, Trans arterial embolization “TAE” through celiac and pancreaticoduodenal arcade failed, likely due to hyper-dynamic circulation-related thrombosis of the proper hepatic artery. That is why we used a novel technique to reach the IAPF with a different method other than TEA.

Overall, TAE is the main management in most children with non-complex congenital IAPF [11, 12, 21, 24, 29, 37, 39, 48] and is curative alone in more than 70% of such cases. After several trials of embolization, a surgical approach should be considered for fistulae that do not resolve [15, 21, 32, 42]. Until sufficient comparative data are available, surgery should be considered as the initial treatment of patients with complex IAPF.

All patients with IAPF after appropriate management have survived except for the first published case (Table 4). Most of the diagnosed children (86.7%, *n* = 39) showed regression of the symptoms, normalization of the portal circulation, preservation of liver functions, and catching up with their growth curves. Only 11% (*n* = 5) of the affected children had persistent portal hypertension [15, 21, 29, 41, 48]. All of them had complex IAPF. Four of them [15, 21, 29, 41] were younger than 4 months and the last one [48] was 7 months, which suggests that younger onset and presentation of complex (type 3) IAPF have a negative effect on prognosis.

**Table 4:** Outcome of patients with IAPF reported in literature

Prognosis	Number of cases (%)
Well and alive	39 (86.7%)
Persistent portal hypertension	5 (11.1%)
Died	1 (2.2%)

## Conclusion

Congenital intra-hepatic arterio-portal fistula is a rare, but treatable cause of portal hypertension in children. Early diagnosis and management will prevent further complications. Any child with recurrent gastrointestinal bleeding, failure to thrive, vomiting, diarrhea, steatorrhea, splenomegaly, or ascites should be investigated for IAPF. Doppler ultrasonography is the first line of investigation in such cases. Treatment modalities depend on the type and size of IAPF as mentioned before. Direct trans hepatic ultrasound guided coiling is a novel technique and an alternative method if TAE failed. Non-complex lesions usually respond to radiological embolization while complex lesions usually need surgical intervention.

## Data Availability

A comprehensive search was done using PUBMED, MEDLINE, GOOGLE SCHOLAR and a reference list of different case reports about congenital intrahepatic arterioportal fistula from the period 1967 to 2020. We reviewed the papers published and ensured that there are no duplicates and that all met the required criteria. (1) Must include pediatric patients, (2) primary diagnosis of included patients was IAPF. Any case that did not meet the criteria was excluded from our search. The full text of each included article was obtained where possible. Summary tables were made for all the extracted papers including gender, type (unilateral, bilateral, and complex), age, symptoms, signs, laboratory investigations, management, and outcome. We collected all this data and started adding it together to reach gender percentage, type percentage, mean age, common symptoms, signs, and investigations. Management was further classified into categories to reach the percentage of patients that underwent each treatment modality and each success rate of all of them. The outcome of all 45 cases reported (including our case) was added together to reach a final prognosis of the IAPF.

## Abbreviations

IHVS: Intrahepaticatic vascular shunts
APF: Arterio-portal fistula
CS: Cesarean section
ASD: Atrial septal defect
PDA: Patent foramen ovale
MSCT: Multi-slice computed tomography
IAPF: Intrahepatic arterio-portal fistula
CHF: Congestive heart failure
AVM: Arteriovenous malformation
TAE: Trans arterial embolization
